# Duration effects of micronutrients in children with ADHD: Randomised controlled trial vs. Open-Label extension

**DOI:** 10.1007/s00787-025-02841-3

**Published:** 2025-09-03

**Authors:** Adarsh Chand, Kathryn Darling, Julia J. Rucklidge

**Affiliations:** School of Psychology, Speech and Hearing, University of Canterbury, Christchurch, New Zealand

**Keywords:** ADHD, Children, Multinutrients, Micronutrients, Open-label, RCT

## Abstract

A 10-week randomised controlled trial (RCT) showed efficacy of micronutrients in improving symptoms associated with Attention-Deficit/Hyperactivity Disorder (ADHD). This study investigated duration effects of micronutrient treatment through the open label (OL) phase and document the micronutrient effect on those initially allocated to placebo. Children aged 7–12 years randomized to micronutrients or placebo for 10 weeks (RCT), then received 10 weeks OL, creating two groups: placebo first then micronutrients (P-M) or micronutrients in both phases (M-M). Assessments included measures of ADHD, emotional dysregulation and Clinical-Global-Impression-Improvement (CGI-I). Of the 93 children enrolled in RCT, 78 (83.9%) completed OL; 37 in P-M and 41 in M-M. For those initially assigned to placebo, CGI-I responders significantly increased from 32.4% in the RCT to 64.9% in OL (*p* = .002); those who took micronutrients for 20 weeks increased from 46.3% (end-of-RCT) to 63.4% responders (end-of-OL) but this was not significant (*p* = .065). Over half of children were treatment responders at end-of-OL, based on 30% reduction in ADHD symptoms from baseline, both from parent (61.5%) and clinician (53.8%) report. Pre-post effect sizes within both groups were significant and very large for all measures, with no significant group differences at end of OL. There were no differences in side effects. Both groups showed significant increases in height over time. This study supports micronutrients as a viable treatment option for ADHD with acute changes maintained and improved over a further 10-week period, with the placebo group catching up to those exposed to micronutrients for full trial duration.

Attention-Deficit/Hyperactivity Disorder (ADHD) is one of the most common mental disorders affecting children, characterised by impairments in attention, hyperactivity and/or impulsivity [1]. If left untreated, individuals are at greater risk for adverse life outcomes, such as anti-social behaviours, poor life quality, substance abuse, and worse academic outcomes [2–4].

Psychostimulants are considered the first-line treatment for ADHD [5]. While medications have been associated with significant short-term improvements in children and adolescents, long-term efficacy is less well documented [6]. Moreover, psychostimulants have been associated with adverse side effects such as appetite loss, reduced height, and weight gain [7–9]. These concerns of adverse effects of medications can lead families to seek alternative treatments, urging research to establish efficacy.

One area of research that has grown over the last decade is the use of a broad-spectrum of micronutrients (vitamins and minerals) as an intervention for ADHD, with three randomised controlled trials (RCTs) showing efficacy in both children and adults with ADHD. These three studies have collectively shown significantly greater change in global improvements of function as well as symptoms of inattention for those taking micronutrients over placebo [10–13]. Specifically for children, Rucklidge et al. [11] showed 47% of those randomised to micronutrients were responders (much to very much improved) compared to 28% of those randomised to placebo based on the Clinical Global Impression - Improvement (CGI-I) rating, over a ten week period. They also documented greater improvements in inattention (but not hyperactivity/impulsivity), aggression and emotional dysregulation in the micronutrient group compared to placebo. Also using the CGI-I, Johnstone et al. [10] found participants taking micronutrients for eight weeks were almost three times as likely to be treatment responders compared with placebo (54% versus 18%), with significant benefits of micronutrients over placebo on inattentive and internalizing symptoms, based on parental report on the Parent Target Problems interview [13] as well as Peer Conflict, as measured by the Child and Adolescent Symptom Inventory-5 (CASI-5). There were no group differences on other target problems such as hyperactivity/impulsivity, autistic symptoms, aggression, or emotional dysregulation/irritable oppositionality. Moreover, longer exposure to micronutrients in the Open Label (OL) phase was associated with significantly more treatment responders in both groups from end of RCT to end of OL [14], with 64% responders for those exposed to micronutrients for 8 weeks and 66% for those exposed for 16 weeks.

The rationale for ingesting a full array of essential micronutrients as a treatment of ADHD symptoms includes providing adequate vitamins and minerals as cofactors to assist enzymes in metabolic functions, improving mitochondrial functioning in the production of adenosine triphosphate (ATP), providing defence against oxidative stress, improving microbiome function, and reducing inflammation [15–17]. In addition, erosion of the modern diet has led to the consumption of more nutrient-poor ultra-processed foods by children [18], reducing their intake of essential vitamins and minerals.

The current study is an extension of the RCT conducted by Rucklidge et al. [11], with an aim to replicate the Leung et al. [14] study of the OL phase. The objectives were to investigate the duration effect of micronutrient treatment over a 20-week period (10 weeks RCT, 10 weeks OL) and to test the micronutrient effect on those who were initially allocated to the placebo group on ADHD symptoms, emotion dysregulation, and overall functioning.

## Methods

### Participants

**Fig. 1:**
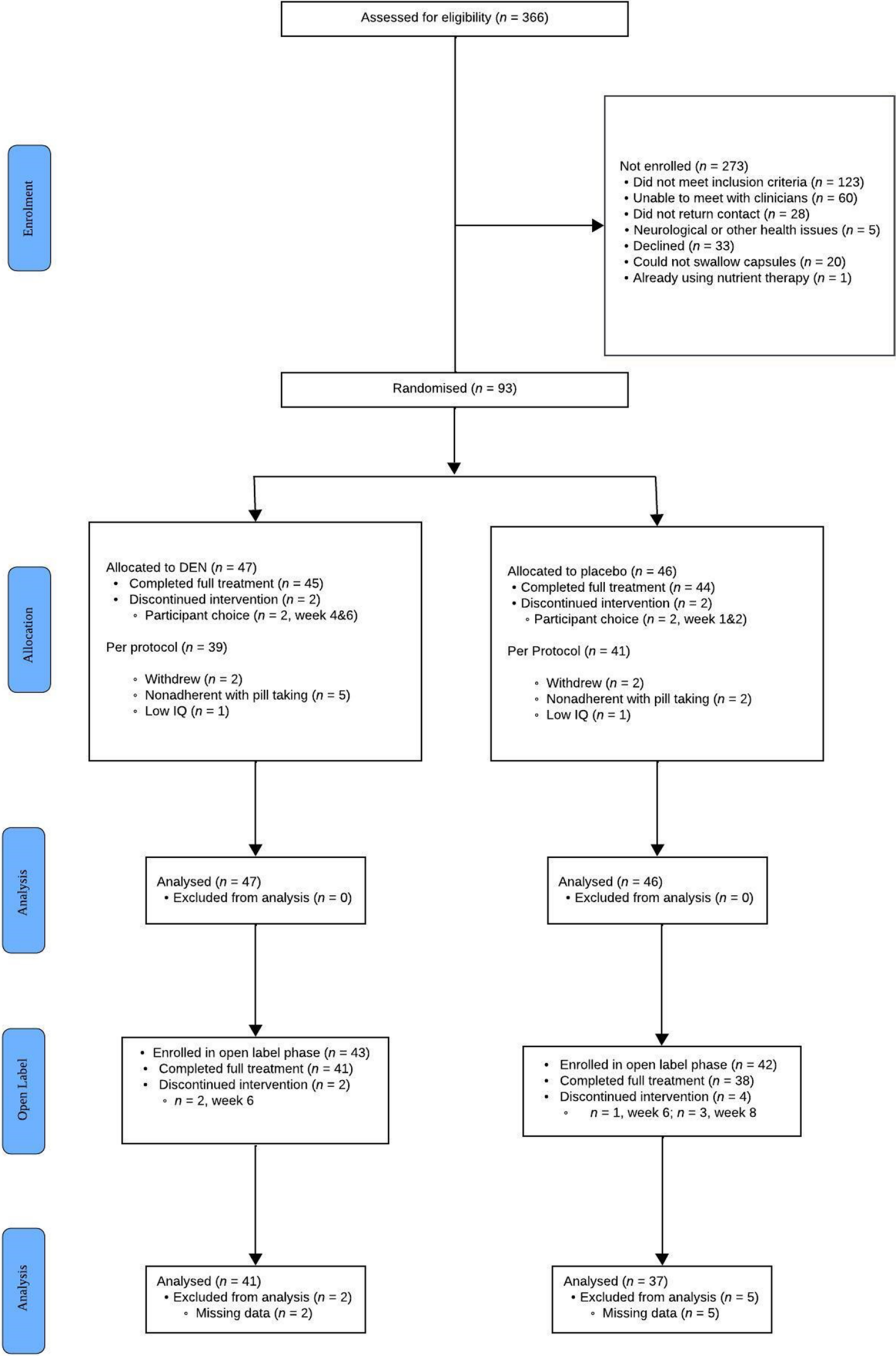
Consolidated Standards of Reporting Trials (CONSORT) Diagram of RCT and OL Phases

Ninety-three children aged 7–12 were initially recruited to a fully blinded, randomised, placebo-controlled trial. Fifteen children either discontinued after the RCT or dropped out before sufficient data could be collected, resulting in 78 (83.9%) participants making up the subsample for this study. Figure 1 presents the flow of the study.

### Procedure

The RCT was carried out for ten weeks, followed by ten weeks of OL (see [11] for further details of procedures). Participants were diagnosed with ADHD using the Schedule for Affective Disorders and Schizophrenia for School-Aged Children – Present and Lifetime Version (K-SADS-PL) interview and elevations on the Conners’ Parent Rating Scale-Revised: Long Version (CPRS: R-L) and Conners’ Teacher Rating Scale-Revised: Long Version (CTRS-L) (T-score greater than 65 for parent and greater than 60 on teacher ratings on one or more of the ADHD subscales). They had to be medication-free and able to ingest up to 15 capsules a day with food. The participants were randomly assigned to micronutrients or placebo in a 1:1 ratio. The micronutrient formula tested was *Daily Essential Nutrients* (DEN), and the placebo group received pills that mimicked the appearance and smell of DEN (they contained a small amount of riboflavin to change colour of urine). Table 1 lists the ingredients for DEN and placebo. The participants started with three capsules daily to take with food and titrated up to 12 capsules daily over a week. After four weeks, participants who did not show substantial improvement based on the CGI-I (a score of three or greater) had their dose increased to 15 capsules daily. Families were provided with a 3-compartment pill box for the three times a day dosing to assist with adherence. Subsequent to this study being conducted, revisions to the formula led to the dose contained within 15 pills now being contained within 12 pills.

**Table 1:** Ingredients of daily essential nutrients and placebo

Daily Essential Nutrients Ingredients: Amount per 12 capsules			
Vitamin A (as retinyl palmiate)	4608 IU	Pantothenic acid (as d-calcium pantothenate)	24 mg
Vitamin C (as ascorbic acid)	480 mg	Calcium (as chelate)	1056 mg
Vitamin D (as cholecalciferol)	2400 IU	Iron (as chelate)	10.8 mg
Vitamin E (as d-alpha tocopheryl succinate)	288 IU	Phosphorus (as chelate)	672 mg
Vitamin K (as menaquinone-7)	24 mcg	Iodine (as chelate)	163.2 mcg
Thiamin (as thiamine mononitrate	48 mg	Magnesium (as chelate)	480 mcg
Riboflavin	14.4 mg	Zinc (as chelate)	38.4 mg
Niacin (as niacinamide)	72 mg	Selenium (as chelate)	163.2 mcg
Vitamin B6 (as pyridozine hydrochloride)	56.4 mg	Copper (as chelate)	6 mg
Folate (as folic acid)	600 mcg	Manganese (as chelate)	7.2 mg
Folate (as L-methylfolate calcium)	600 mcg	Chromium (as chelate)	499.2 mcg
Vitamin B12 (as methylcobalamin)	720 mcg	Molybdenum (as chelate)	115.2 mcg
Biotin	864 mcg	Potassium (as chelate)	192 mg
Proprietary blend: Choline bitartrate, alpha-lipoic acid, inositol, acetyl-L-carnitine, grape seed extract, ginkgo biloba leaf extract, L-methionine, N-acetyl-L-cysteine, chelated trace minerals: germanium sesquioxide, boron, vanadium, lithium orotate, nickel. Other ingredients: Gelatin, cellulose, glycine, citric acid, magnesium stearate, silicon dioxide, mineral wax, titanium dioxide.			
**Placebo Ingredients**: Amount per 12 capsules			
Fiber Acacia Gum	3600 mg	Cocoa powder	48 mg
Maltodextrin	4750.8 mg	Riboflavin powder	1.2 mg

Participants were typically monitored in person every two weeks, resulting in 11 total visits over 20 weeks. The fortnightly visits usually lasted 20–30 min, while visits at switch points (end of RCT and end of OL) lasted 45–60 min, as they included assessments of additional measures. The participants were assessed by a clinical psychologist or graduate student under the supervision of a psychologist. Five per cent of the assessments were conducted by phone, for those few participants who lived outside of Christchurch. Each participant was assigned to one monitoring clinician to assist with the reliability of ratings over time. Further, all clinicians were overseen by the same clinical psychologist to ensure consistency in ratings across different clinicians.

The clinicians, parents, children, and teachers completed measures relating to ADHD symptoms, emotion regulation, and psychological behaviours at all visits. Families were given a new bottle of capsules at each assessment. Adherence during the OL phase was determined by parental reports of the number of doses missed in the previous two weeks.

In the OL phase, all participants received the micronutrients; however, the RCT assignments were kept blind. Everyone who had contact with the families and the families themselves in the OL did not learn of participants’ assignments until the RCT data analyses were completed.

### Clinician-rated measures

1. The Clinical Global Impressions-Improvement-Global scale (CGI-I) [19] was used to capture overall improvements. The clinicians considered parent verbal reports, changes in rating questionnaires, information from teachers and behaviour in the clinic to assist with the rating. These measures span from 1 (very much improved) to 7 (very much worse).
2. The Children’s Global Assessment Scale (C-GAS) [20] captured the child’s overall social and psychiatric functioning on a scale from 1 to 100, where a higher score indicated better functioning.
3. The ADHD Rating Scale-IV (ADHD-RS-IV) is an 18-item scale about ADHD symptoms linked to the DSM-5 diagnostic criteria for ADHD [21]. The clinicians used information from parent verbal reports, information from teachers and behaviour in the clinic to rate participants. The scale is based on frequency of symptoms observed, ranging from ‘0, Never’ (symptom occurred once a week or less) to ‘3, Very Often’ (symptom occurred every day or multiple times a day).
4. Height was also measured at all on-site visits.

### Parent-rated measures

1. The Child Mania Rating Scale, Parent Version (CMRS-P) [22] was used to assess the symptoms of feeling irritable, racing thoughts, rage attacks and rapid mood swings. The items were rated from 0 (never/rarely) to 3 (very often). The total score on the scale ranges from 0 to 63. Scores over 20 indicated a child being at risk for severe mood dysregulation.
2. The Conners’ Parent Rating Scale-Revised: Long Version (CPRS-R: L) [23] asks the parent to reflect on the child’s behaviour over the previous month. It has a four-point Likert scale where 0 was ‘not very true at all’ to 3, which was ‘very much true.’ Only three subscales (inattention, hyperactivity, and DSM total) were analysed, as they are directly related to ADHD.

#### Statistical analysis

All statistical analyses were conducted using SPSS IBM^®^ Statistics 29. Violations of normality were checked using Shapiro-Wilk tests and Q-Q plots. Cook’s distance scores were used to identify any outliers. The threshold (4÷*n*) was used, therefore, any values greater than 0.05 were flagged as potentially influential. Additionally, Cook’s D values greater than one were further inspected. If any outliers were identified, the analysis was run with and without it. The original model was reported if there was no statistical difference between the two models.

Independent samples *t*-tests were conducted for continuous variables, and chi-square analyses were conducted for dichotomous variables to compare sociodemographic characteristics between those who did and did not complete OL. Fisher’s exact test was used if more than 20% of the cells had expected frequencies of less than five.

Since this study used a subsample of the original study, the RCT results for the subsample were examined in addition to the OL results. The group that received micronutrients in both RCT and OL was named Micronutrient-Micronutrient (M-M). The group that received a placebo in the RCT and micronutrients in OL was named Placebo-Micronutrient (P-M). Repeated measures ANOVA was used to test the duration effect within and between groups (baseline, week 10, and week 20) on all primary outcome measures and height data. Between-group changes from baseline to week 20 were compared using an ANCOVA, with age as a covariate. Chi-square tests were used to compare categorical outcomes between groups at week 20. However, Fisher’s exact test was used if more than 20% of the cells had expected frequencies of less than five. Cohen’s d was used to measure effect size (0.20 small, 0.50 medium, and 0.80 large effect size).

McNemar’s test was used to identify any within-group differences in treatment responders at week 10 and week 20. The treatment effects were summarised by mean differences with 95% confidence intervals derived from all models. All models with p-values < 0.05 were considered statistically significant.

CGI-I-Global Impression identified treatment responders as either ‘much’ or ‘very much improved’ on improvement across all areas of functioning. Also, a 30% decrease on the ADHD-RS-IV and CPRS-R: L scales compared with baseline identified that ADHD symptoms had decreased by a clinically meaningful amount, thus indicating a treatment responder. This percentage change is often used in the literature to identify meaningful responses [24].

## Results

### Demographics

Of the 93 participants in the initial study, 15 (16.1%) stopped after the RCT. There was a higher prevalence of children with divorced parents continuing OL (10.3%) compared to those participants who stopped after RCT (0%). No other group differences were noted. Table 2 presents the means and frequencies for all demographic characteristics comparing those who completed and did not complete OL.

**Table 2:** Sociodemographic characteristics of the participants and parents of those who completed open label (OL) and those who stopped after randomised controlled trial (RCT) represented by mean, standard deviation and sample percentage

	Groups		
	Completed OL *N* = 78	Stopped After RCT *N* = 15	*p* value
Age	9.59 (1.47)	10.13 (1.77)	0.21
Sex:			1.00^a^
Male	60 (76.9%)	12 (80.0%)	
Female	18 (23.1%)	3 (20.0%)	
Ethnicity^b^			.31^a^
NZ European	68 (87.2%)	14 (93.3%)	
NZ Māori	5 (6.41%)	0	
Tongan	0	1 (6.6%)	
European	2 (2.6%)	0	
Other	2 (2.6%)	0	
Screening ADHD Rating Scale-IV			
Inattention	21.50 (3.82)	22.27 (2.60	0.46
Hyperactivity	20.51 (5.11)	19.80 (5.86)	0.66
ADHD Total	42.01 (7.54)	42.07 (7.09)	0.98
Parent Marital Status			0.05^a^
Married	48 (61.5%)	6 (40.0%)	
De facto	10 (12.8%)	7 (46.7%)	
Single	10 (12.8%)	2 (13.3%)	
Divorced	8 (10.3%)	0	
Widow	2 (2.6%)	0	
NZSEI Score^b^	46.45 (15.10)	53.07 (14.78)	0.12

NZSEI = New Zealand Socioeconomic Index, ^a^Fishers Exact Test, ^b^1 missing ethnicity, 5 NZSEI scores missing. The range of scores for ADHD Rating Scale-IV inattention and hyperactivity: 0–27, respectively, and 0–54 for ADHD Total

Of the 78 participants remaining in OL, 41 were M-M, and 37 P-M. There was a significant difference in age between the M-M and P-M groups (*t*(76)= −2.29, *p =*.03), with the M-M group slightly older (9.94 years) than the P-M group (9.19 years). No other significant differences in characteristics were noted between groups (Table 3). Note that 14 of the M-M group and 7 of the P-M group had a past history of taking stimulant medications. The rest of the sample was medication naïve.

**Table 3:** Sociodemographic characteristics of the participants and parents in the Micronutrients – Micronutrients (M-M) and Placebo – Micronutrients (P-M) groups represented by mean, standard deviation and sample percentage

	Groups		
	M – M (*N* = 41)	P – M (*N* = 37)	*p* value
Age	9.94 (1.33)	9.19 (1.52)	0.03
Sex			0.77
Male	31 (75.6%)	29 (78.4%)	
Female	10 (24.4%)	8 (21.6%)	
Ethnicity^b^			0.54 ^a^
NZ European	37 (90.2%)	31 (83.7%)	
NZ Māori	1 (2.4%)	4 (10.9%)	
European	1 (2.4%)	1 (2.7%)	
Other	1 (2.4%)	1 (2.7%)	
Screening ADHD			
Inattention	21.71 (3.89)	21.27 (3.78)	0.62
Hyperactivity	20.68 (4.71)	20.32 (5.57)	0.76
ADHD Total	42.39 (6.92)	41.59 (8.25)	0.65
Parent Marital Status			.82^a^
Married	27 (65.9%)	21 (56.8%)	
De facto	4 (9.8%)	6 (16.2%)	
Single	4 (9.8%)	6 (16.2%)	
Divorced	5 (12.2%)	3 (8.1%)	
Widow	1 (2.4%)	1 (2.7%)	
NZSEI Score^b^	46.29 (14.31)	46.63 (16.13)	0.92

^a^Fishers Exact Test, ^b^1 missing ethnicity, 5 NZSEI scores missing, NZSEI = New Zealand Socioeconomic Index. The range of scores for ADHD Rating Scale-IV inattention and hyperactivity: 0–27, respectively, and 0–54 for ADHD Total

### Clinician rated CGI-I-Global

The mean change from week 10 to week 20 was significant for the P-M group (∆ 0.81, 95% CI 0.42, 1.20), *t*(36) = 4.20, *p* <.001; *d* = 0.69, and the M-M group (∆ 0.51, 95% CI 0.15, 0.87), *t*(40) = 2.87, *p =*.007; *d =* 0.45. There were no significant group differences at week 20, *F*(1, 77) = 3.29, *p =*.074, ηp^2^ = 0.04. Table 4 displays the means and changes on all scales for both groups.

**Fig. 2:**
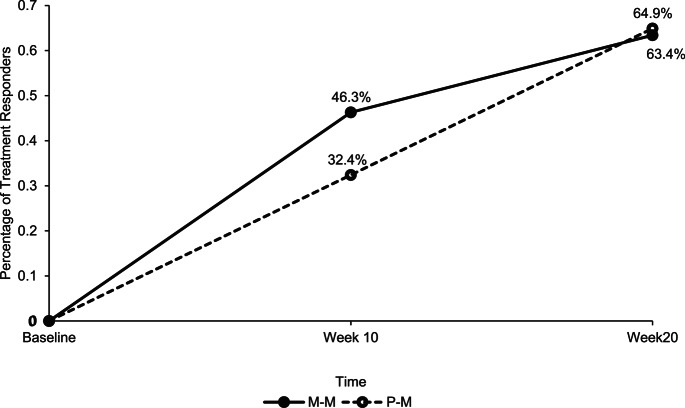
Percentage of treatment responders between the P-M and M-M groups based on the CGI-I-Global Scale at Week 10 and Week 20. *M–M = Micronutrient–Micronutrient*,* P–M = Placebo–Micronutrient*.

There was a significant increase in treatment responders from week 10 (32.4%) to week 20 (64.9%), *p* =.002 for the P-M group. The M-M group also saw an increase in treatment responders from week 10 (46.3%) to week 20 (63.4%); however, this change was not significant, *p* =.065 (see Fig. 2). There was no group difference in the number of treatment responders at week 20, *χ*^*2*^ (1, *N =* 78) = 0.02, *p* =.89.

**Table 4:** Means and standard deviations for all outcomes at all time points and within and between group comparisons across time

P-M (*n* = 37)				Within Group Change				M-M (*n* = 41)			Within Group Change			Between group Change^a^ (*p*)
	Baseline	Week 10	Week 20		Baseline to Week 10	Week 10 to Week 20	Week 0 to Week 20	Baseline	Week 10	Week 20	Baseline to Week 10	Week 10 to Week 20	Baseline to Week 20	Baseline to Week 20
CPRS: R-L														
Inattention	22.03 (3.75)	17.89 (5.84)	15.62 (6.87)		−4.14**	−2.27*	−6.41**	21.95 (4.38)	17.78 (5.95)	14.54 (6.00)	−4.17**	−3.24**	−7.41**	0.55
Hyperactivity	20.70 (4.74)	16.38 (6.38)	13.81 (6.81)		−4.32**	−2.57*	−6.89**	20.59 (4.02)	15.78 (6.30)	13.05 (6.73)	−4.81**	−2.73*	−7.54**	0.39
DSM Total	42.73 (7.10)	34.27 (11.17)	29.43 (12.87)		−8.46**	−4.84*	−13.30**	42.59 (6.72)	33.32 (10.76)	26.90 (10.80)	−9.27**	−6.42**	−15.68**	0.43
ADHD-RS-IV														
Inattention	23.70 (2.68)	21.43 (4.85)	17.73 (5.69)		−2.27*	−3.70**	−5.97**	24.12 (3.09)	19.95 (6.13)	16.00 (5.98)	−4.17**	−3.95**	−8.12**	0.55
Hyperactivity	21.84 (4.44)	17.22 (6.67)	14.03 (6.60)		−4.62**	−3.19*	−7.81**	20.93 (5.54)	17.44 (6.81)	13.02 (6.76)	−3.49**	−4.41**	−7.90**	0.84
Total	45.54 (5.45)	38.65 (10.08)	31.76 (10.66)		−6.89**	−6.89**	−13.78**	45.05 (6.84)	37.39 (11.25)	29.02 (11.18)	−7.66**	−8.37**	−16.02**	0.61
CMRS-P	24.51 (11.54)	17.38 (11.90)	13.35 (10.15)		−7.14**	−4.03**	−11.16**	25.34 (11.18)	14.71 (10.32)	10.73 (7.46)	−10.63**	−3.98**	−14.61**	0.25
C-GAS	48.43 (6.74)	51.76 (9.43)	58.30 (9.05)		3.32*	6.54**	9.87**	48.61 (6.54)	54.29 (9.57)	60.12 (10.09)	5.68**	5.83**	11.51**	0.63
CGI-I Global	-	3.16 (0.93)	2.35 (1.11)		-	0.81**	-	-	2.80 (1.12)	2.29 (1.01)	-	0.51*	-	-

M–M = Micronutrient–Micronutrient, P–M = Placebo–Micronutrient, CPRS:-L = Conners’ Parent Rating Scale-Revised: Long Version; ADHD-RS-IV = ADHD Rating Scale-IV; CMRS-P = Child Mania Rating Scale (scores range from 0–63), Parent Version; C-GAS = Children’s Global Assessment Scale (scores range from 1-100 with a lower score more indicative of greater functional impairment); CGI-I-Global = Clinical Global Impressions-Improvement Scale (ranging from 1 (very much improved) to 7 (very much worse)), CGI-I-Global was not measured at baseline, therefore only week ten and week twenty results were compared. CPRS: R-L/ADHD-RS-IV inattention: range: 0–27, hyperactivity: range: 0–27, and DSM total: range: 0–54. *<0.05, **<0.01, ^a^Between group change calculated as change score P-M (Week 20 – baseline) compared to change score M-M (Week 20 – baseline)

### CGAS

A significant main effect of time was found on CGAS scores across the three-time points, *F*(1.85, 140.77) = 78.98, *p* <.001, ηp^2^ = 0.51. There was no interaction effect between time and group, *F*(1.85, 140.77) = 1.00, *p =*.37, ηp^2^ = 0.01. Participants who took the micronutrients for ten weeks showed continuous improvement on the CGAS scores from baseline to week 20 (∆ 9.87, 95%CI 7.26, 12.47, *p* <.001, *d =* 1.43). The participants who took the micronutrients for twenty weeks improved on the CGAS scores from baseline to week 20 (∆ 11.51, 95% CI 9.04, 13.98, *p <*.001, *d =* 1.31). CGAS scores at week 20 were not significantly different between the P-M and M-M groups.

#### Clinician-rated ADHD-RS-IV

The main effect of time was significant on inattention scores, *F*(2, 152) = 62.92, *p <*.001, ηp^2^ = 0.45, hyperactivity scores, *F*(2, 152) = 80.18, *p <*.001, ηp^2^ = 0.51, and on ADHD-Total scores, *F*(2, 152) = 86.04, *p* <.001, ηp^2^ = 0.53. However, there were no significant interaction effect between time and group for these three scores. Participants in the P-M group improved on all three subscales from baseline: [Inattention (∆ −5.97, 95% CI – 7.89, − 4.05, *p* <.001, *d =* 1.09); Hyperactivity (∆ − 7.81, 95% CI − 9.80, − 5.82, *p* <.001, *d =* 1.20); ADHD-total (∆ − 13.78, 95% CI −17.35, − 10.22, *p* <.001, *d =* 1.25)]. Similarly, participants who took the micronutrients for twenty weeks (M-M) improved on all three subscales from baseline: [Inattention (∆ − 8.12, 95% CI – 9.95, − 6.30, *p* <.001, *d =* 1.31); Hyperactivity (∆ − 7.90, 95% CI − 9.79, − 6.02, *p* <.001, *d =* 1.41); Total (∆ − 16.02, 95% CI − 19.41, − 12.64, *p* <.001, *d =* 1.49)]. No group differences were found between P-M and M-M at week 20.

**Fig. 3:**
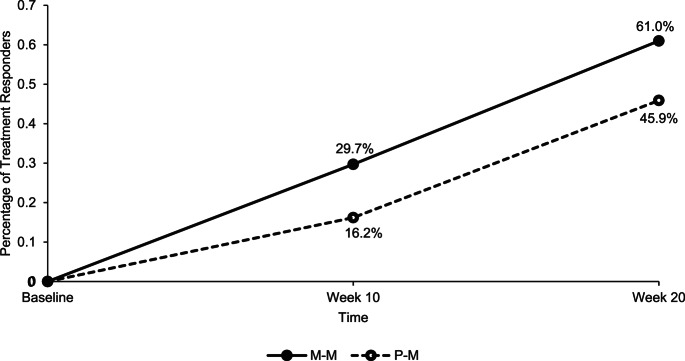
Percentage of Treatment Responders (defined as 30% Reduction in ADHD Symptoms compared to baseline) in the M-M and P-M group based on the Clinician-rated ADHD-RS-IV scale at Week 10 and 20. M–M=Micronutrient–Micronutrient, P–M=Placebo–Micronutrient

Participants in the P-M group saw an increase in treatment responders from 10.8% at end of RCT to 40.5% at end of OL (*p =*.007) on the inattention subscale, 27.0–59.5% (*p =*.004) on hyperactivity, and 16.2–45.9% (*p =*.007) on ADHD-total, based on 30% decrease in ADHD-RS-IV scores relative to baseline. Similarly, there was a significant increase in treatment responders in the M-M group based on the 30% decrease in ADHD-RS-IV scores. Treatment responders based on the inattention subscale increased from 31.7% (end of RCT) to 58.5% (end of OL) (*p =*.003), 24.4–65.9% (*p <*.001) on hyperactivity, and 29.7–61.0% (*p <*.001) based on ADHD-total scores. There were no significant differences in the number of treatment responders at the end of OL between the groups (P-M and M-M) in the number of treatment responders for inattention, *χ*^*2*^ (1, *n =* 78) = 2.52, *p =*.11, hyperactivity, *χ*^*2*^ (1, *n* = 78) = 0.34, *p* =.56, and ADHD-total, *χ*^2^ (1, *n* = 78) = 1.77, *p =*.18. Figure 3 presents percent treatment responders for the two groups on the total ADHD scale.

The main effect of time was significant on inattention scores, *F*(2, 152) = 55.49, *p <*.001, ηp^2^ = 0.42, hyperactivity scores, *F*(2, 152) = 61.21, *p <*.001, ηp^2^ = 0.45, and DSM-IV scores, *F*(2, 152) = 72.15, *p <*.001, ηp^2^ = 0.49. However, there was no significant interaction effect between time and group on any of these subscales.

Participants in the P-M group improved on all three subscales from baseline: [Inattention (∆ −6.41, 95%CI − 8.46, − 4.35, *p* <.001, *d* = 0.96); Hyperactivity (∆ − 6.89, 95%CI – 8.95, − 4.84, *p* <.001, *d =* 1.07); DSM-IV Total (∆ − 13.30, 95%CI −17.15, − 9.44, *p* <.001, *d* = 1.07)]. Similarly, participants who took the micronutrients for twenty weeks (M-M) improved on all three subscales from baseline: [Inattention (∆ − 7.42, 95%CI − 9.37, − 5.46, *p* <.001, *d* = 1.25); Hyperactivity (∆ − 7.54, 95% CI − 9.49, − 5.59, *p* <.001, *d* = 1.23); DSM-IV Total (∆ − 15.68, 95%CI − 19.35, − 12.01, *p* <.001, *d =* 1.41)]. No group differences were found between P-M and M-M at end of OL on inattention, hyperactivity, and DSM-IV total.

**Fig. 4:**
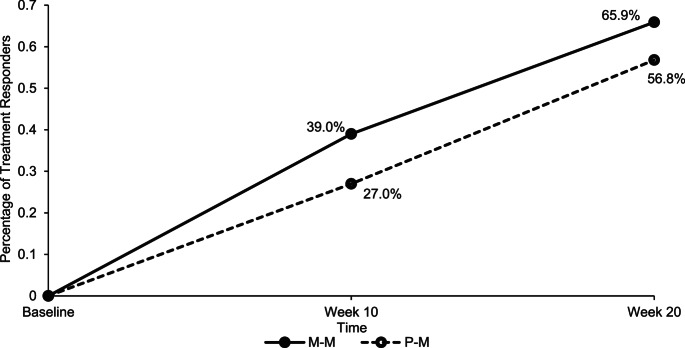
The Percentage of Treatment Responders (defined as 30% Reduction in ADHD Symptoms compared to baseline) for the M-M and P-M groups on the Parent-rated CRPS: R-L Scale at Week 10 and Week 20. M–M = Micronutrient–Micronutrient, P–M = Placebo–Micronutrient

The number of treatment responders in the P-M group significantly increased from 27% at week 10 to 56.8% at week 20 (*p =*.007), based on a 30% reduction in the CPRS-:L scores from baseline. The M-M group saw a significant increase in treatment responders, from 39% at week 10 to 65.9% at week 20 (*p =*.003). There were no significant differences in treatment responders between groups at week 20, *χ*^*2*^ (1, *N =* 78) = 0.68, *p* =.41 (see Fig. 4).

#### Parent-rated CMRS

A significant effect of time was found, *F*(1.65, 125.01) = 78.32, *p <*.001, ηp^2^ = 0.51, but no significant interaction effect between time and group, *F*(1.65, 125.01) = 1.81, *p =*.17, ηp^2^ = 0.02. Both participants in the P-M group showed improvement from baseline to week 20 (∆ − 11.16, 95% CI – 14.57, − 7.75, *p <*.001, *d =* 1.15), as well as participants in the M-M group (∆ − 14.61, 95% CI – 17.85, − 11.37, *p <*.001, *d =* 1.33). There were no significant group differences at week 20. Considering only those who had a score > 20 at baseline (identifying those with moderate mood problems), 20 out of 30 (66.7%) of the P-M group went into remission (dropping below 20) and 26 out of 32 (81.3%) of those in the M-M group went into remission.

### Height

**Fig. 5:**
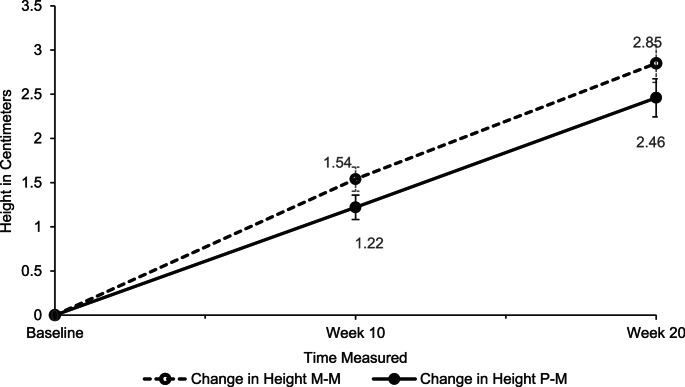
Height Increase in Centimetres from Baseline to Week 10 and Week 20. M–M = Micronutrient–Micronutrient, P–M = Placebo–Micronutrient.

Two participants in the M-M group had missing data and were excluded from the analysis (P-M: 37 and M-M: 39). A significant effect of time was shown on height from Baseline to Week 20, *F*(1.55, 114.73) = 172.37, *p <*.001, ηp^2^ = 0.70. The P-M group had a height increase of 1.22 centimetres (cm) after ten weeks, and 2.46 cm increase at twenty weeks, compared to baseline. The M-M group had a height increase of 1.54 cm after ten weeks and a 2.85 cm increase at twenty weeks. No significant group differences were identified across the three time points, *F*(1, 74) = 2.69, *p* =.105, ηp^2^ = 0.04 (See Fig. 5). No significant height differences were observed between participants who had and had not been exposed to stimulants prior to the study in either group, across three time points. Those who were medication naïve (*n* = 55) showed a similar trajectory in height as shown in Fig. 5.

#### Adherence and Treatment-emergent side effects

Based on parental-report, adherence was excellent, with 94.2% (SD = 5.14) of doses reported to have been taken. There were no group differences in emerging adverse events during the OL phase: 33 in the P-M group and 26 in the M-M group. Rates of the more common adverse events (AEs) (Table 5) were generally low. 64% of reported AEs resolved on their own, and the others required a dose adjustment to resolve the side effect. Only two of the AEs (headaches and gastrointestinal disturbances) were identified as definitely related to the treatment. No serious or severe adverse events were reported, and only 1 of the 59 adverse events reported was rated as moderate.

**Table 5:** Treatment-Emergent adverse events during the Open-Label phase

	P-M (*n* = 37) *n* (%)	M-M (*n* = 41) *n* (%)
Headache	5 (13.5%)	3 (7.3%)
Dry mouth	0	2 (4.9%)
Sleep disruptions	5 (13.5%)	2 (4.9%)
Gastrointestinal disturbances	5 (13.5%)	7 (17.1%)
Nausea	2 (5.4%)	2 (4.9%)
Irritability	1 (2.7%)	1 (2.4%)
Fatigue	2 (5.4%)	3 (7.3%)
Anxiety	4 (10.8%)	2 (4.9%)
Eating problems	2 (5.4%)	0
Rash	3 (8.1%)	2 (4.9%)
Bedwetting	0	0
Suicidal thoughts	2 (5.4%)	0
Nosebleed	0	0
Migraine	0	0

*M–M * Micronutrient–Micronutrient, *P–M* Placebo–Micronutrient

## Discussion

The current study extended the findings of Rucklidge et al. [11] by comparing the RCT to the OL phase and was consistent with reports from the Micronutrients for Attention-Deficit/Hyperactivity Disorder in Youth (MADDY) study on benefits observed at the end of OL [14]. We investigated the changes in primary outcomes and treatment responders for children who took micronutrients for 20 weeks compared to children who took them for 10 weeks.

The results demonstrated that micronutrient supplementation significantly improved ADHD symptoms, emotional regulation (with 74% of those identified as moderately dysregulated at baseline going into remission), and overall functioning across all parent- and clinician-rated scales, regardless of whether participants received micronutrients for ten or twenty weeks, with large within-group effect sizes for both groups (*d =* 0.96–1.49). By week 20, there were no group differences on any of the outcomes, showing that the placebo group caught up in changes observed in the micronutrient group during RCT.

Twenty weeks of micronutrient supplementation could support maintenance and continual improvement of ADHD symptoms. This is consistent with the MADDY study, which reported significant improvements in ADHD symptoms based on clinician and parent-rated scales [25]. Similarly, studies exceeding the 20-week duration have recorded significant improvements in ADHD symptoms from baseline [26–28]. Darling et al. [26] noted participants who had better outcomes on the psychological measures at end of the OL (20 weeks) were more likely to continue using micronutrients in the follow-up instead of switching to psychiatric medication or discontinuing treatment altogether. This suggests that a 20-week micronutrient intervention may be sufficient for families to observe benefits, potentially fostering greater adherence to long-term treatment.

The results showed that twenty weeks of micronutrient supplementation resulted in nearly two-thirds of the M-M group being classified as treatment responders based on a 30% reduction in ADHD symptoms, both on the clinician and parent-rated ADHD scales. The CGI-I demonstrated similar percentages of treatment responders. The beneficial effects observed for those who were moderately dysregulated at baseline should also be a welcomed change given the challenges of improving irritability and temper explosions in children and young people [29]. Notably, the delayed introduction of micronutrients in the P-M group led to a comparable and significant increase in treatment responders.

Both groups showed significant height increases compared to baseline. Similar to Leung et al. [14], the M-M group displayed a trend for greater height change than the P-M group, but no significant differences between groups were observed. Similar to the MADDY study [10], we did not observe an interaction between those who had a history of being on stimulant medications and those who hadn’t, suggesting that the change in height is indicative of typical growth alongside a potentially small boost from the micronutrients and not simply a reflection of rebound growth that can happen when stimulant medications are discontinued [30]. The reported benefits of micronutrients on height contrast with negative effects of ADHD stimulant medications on growth velocity. Children with ADHD show slowed growth velocity relative to children not medicated during the first six months after starting medication, with younger children and those experiencing moderate to severe appetite loss at a greater risk of these height restrictions [31].

In line with open-label extensions and longer-term follow-up trials (0.31–5 years), our study showed low rates of adverse events, with no group differences in emerging side effects. This demonstrated that micronutrients have a safe profile for extended durations [14, 32, 33]. Headaches and gastrointestinal disruptions were common side effects, but these were usually mitigated by taking the capsules with a full stomach and drinking plenty of water [33]. Micronutrients also have a safer profile compared to stimulant medications, which have been associated with more appetite suppression and insomnia [34, 35].

The study had several strengths. The study had a high retention rate (83.4%) from the end of the RCT to the open-label extension phase. Another strength is that the participants, clinicians, and parents were blinded to the RCT assignment until the end of the OL trial, potentially minimising bias and improved validity. The diagnostic procedures reflected gold standard methodology, assessing ADHD across multiple settings. Moreover, the study yielded large within-group effect sizes. Another strength is that all participants were medication-free during the 4 month trial, allowing for observations of potential effects of micronutrients as the sole intervention for ADHD.

All participants in the study were aware they were taking micronutrients during the OL phase. This could have introduced biases such as expectancy effects, respondent bias and social desirability bias. Furthermore, the P-M group significantly improved on primary outcome measures during the RCT phase, indicating a strong placebo effect, likely influenced by expectancy and the frequent clinician contacts and care they received as part of the clinical trial. Reliance on retrospective reporting on ADHD symptoms from parents could have introduced biases such as recency effects and saliency of behaviour, which can affect report accuracy [36]. Clinician-rated measures are likely more reliable because they are less likely to be influenced by these biases, as clinicians are trained to assess behavioural changes and integrate parent and teacher reports into their assessments [11], suggesting greater weight should be extended to the clinician measures. Another limitation is that the trial included numerous assessment points, which introduced testing and maturation effects. Fourteen participants dropped out of the open-label phase, which could have increased or decreased the percentage of treatment responders over time. Lastly, the sample was predominantly male and lacked ethnic diversity, which could affect the generalisability of the results to other populations.

## Conclusion

This study demonstrated that micronutrient treatment improved the symptoms of ADHD, emotional regulation and overall functioning from end of RCT to end of OL. More importantly, those children who were exposed to micronutrients for a longer duration continued to show improvements in ADHD symptoms. The most notable improvement was noticed in the ADHD-RS-IV scale, where a longer duration of micronutrient exposure resulted in significantly more participants experiencing a 30% or more reduction in core ADHD symptoms (inattention, hyperactivity, and ADHD total). Future studies would benefit from using longer follow-up designs in a naturalistic setting to capture the treatment effect outside of the controlled trial conditions. With two independent child RCTs both showing clinically meaningful benefit in ADHD and associated symptoms alongside minor side effects, with about two thirds identified as responders to the micronutrients after four months, this intervention should be considered for standard clinical use in paediatric ADHD treatment.

## Data Availability

No datasets were generated or analysed during the current study.
